# Epidemiological evaluation of human papillomavirus genotypes and their associations in multiple infections

**DOI:** 10.1017/S0950268818003539

**Published:** 2019-03-01

**Authors:** Raffaele Del Prete, Luigi Ronga, Raffaella Magrone, Grazia Addati, Angela Abbasciano, Domenico Di Carlo, Giuseppe Miragliotta

**Affiliations:** 1 Section of Microbiology, Interdisciplinary Department of Medicine (DIM), School of Medicine, University of Bari ‘Aldo Moro’, Bari, Italy; 2 UOC Microbiology and Virology, Azienda Ospedaliera-Universitaria Policlinico of Bari, Bari, Italy; 3 Pediatric Clinical Research Center ‘Romeo and Erica Invernizzi’, University of Milan, Milan, Italy; 4 Department of Biology and Biotechnology, University of Pavia, Pavia, Italy

**Keywords:** Epidemiology, multiple HPV-types infections, multiplex real-time PCR, nested PCR

## Abstract

The aim of this study was to determine the frequency of multiple type human papillomavirus (HPV) infections, and whether any types are involved in multiple HPV-type infections (mHPV) more or less frequently than expected. From January 2012 to February 2018, 2848 cervico-vaginal swabs were analysed in the UOC Microbiology and Virology of Policlinico of Bari, Italy. HPV DNA detection was performed using initially nested-polymerase chain reaction (PCR) and subsequently multiplex real-time PCR assay. 1357/2848 samples (47.65%) were HPV DNA positive and 694/1357 (51.14%) showed mHPVs. The median number of mHPVs was 2 (interquartile range: 2–3). HPV-types more frequently detected were 42 (9.97%), 16 (8.92%), 53 (7.23%) and 31 (7.16%). Each detected HPV-type was involved in mHPVs in more than 50% of cases. Statistical analysis showed significant associations for all HPV-types except for 33, 43, 51, 58 and 82 HPV-types. The major number of significant pairwise associations were detected for the types 42 and 70. Only positive associations were detected. Further data are necessary to evaluate the clinical impact of the single combinations.

## Introduction

Human papillomavirus (HPV) infection contributes to the burden of diseases as genital warts and cancer, in both men and women [1–3]. In particular, nowadays, different studies have demonstrated that there is a high incidence of cervical cancer (CC) by a co-infection of different HPV-types. Thus, HPV co-infections might be considered a risk factor for the carcinogenesis, although different studies reached different conclusions, in which a correlation between multiple HPV-type (mHPV) infections and the carcinogenesis is not always demonstrated [4, 5]. Moreover, it is not clear if the risk for the progression to cancer is correlated to some HPV-type specific combinations or if it is common for all the genotypes [6–10]. Some infections, however, may persist for many years, causing cellular alterations, which, if untreated, have the potential to develop into cancer of ano-genital area (carcinoma of the cervix, vagina, vulva, anal, oral and penile areas) [11, 12].

Recently, it has been reported that mHPV infections are apparently more widespread than expected [13, 14]. Although the genital infections with mHPVs are common in either men or women [1, 15–17], the relative rates might not be representative for the prevalence due to the limited number of patients studied [13, 14, 18]. Nevertheless, since when the HPV-types have been performed by polymerase chain reaction (PCR) assays, mHPV infections have been more consistently described [19]. Among the studies, relevant differences in mHPV prevalences have been described. For example, Dickson *et al*. have reported that in large studies performed in Costa Rica and Italy, the mHPV infection prevalence range from 24.8% to 52.6% among all HPV-positive tests [5]. Such differences may be explained by the genotyping techniques used and also by the type of and behavioural characteristics of the populations analysed. Furthermore, epidemiological studies have found that patients infected with mHPVs require a management to prevent the progression for cancer, underlining that the monitoring of the genotypes and of the viral load might provide some critical information in the diagnosis of infections either for future management or prognosis [20–22]. Nevertheless, the relationship between mHPVs and CC is still debatable.

In the past several studies had attempted to assess patterns of mHPVs in women [1, 5, 20]. However, different statistical models had been used to analyse the data, obtaining results either different or hardly comparable.

Moreover, mHPVs were seldom detected using earliest diagnostic methods and only the shift towards more sensitive assays allowed confirming that mHPVs are either highly widespread or very common among sexually active women [19]. Moreover, some authors suggested that the apparent clustering of mHPVs may be an artefact due to the measurement process (i.e. diagnostic assay) raising the question of the cross-reactions as confounding factor [1].

The aim of this study was to determine the prevalence of mHPVs in a cohort of women and also evaluate the association of pairwise HPV-types accounting for the analytical genotyping method.

## Methods

### Study population

From January 2012 to February 2018, 2848 cervico-vaginal swabs from 2848 women were consecutively collected. In the case of multiple samples from the same patient, only the first was retained for the analysis. Median age of women analysed was 37 years. The specimens were transferred to the laboratory of Molecular Biology, UOC Microbiology and Virology, Azienda Universitaria-Ospedaliera, Policlinico of Bari, where they were analysed.

All procedures performed in studies involving human participants were in accordance with the 305 ethical standards of the institutional and/or national research committee and with the 1964 Helsinki declaration and its later amendments or comparable ethical standards. Sample information (date of sampling, ward, type of specimen, testing results) together with the data of patients for whom molecular testing was performed (i.e. age and sex) were recorded in an anonymous database by changing sensitive data into alphanumeric codes. No clinical data associated with these specimens were available. The study was approved by Ethical Committee of Policlinico of Bari (No. 0022174|12|03|2018).

### Treatment of specimens

In total 2 ml of phosphate-buffered saline (pH 7.4) (Sigma-Aldrich, Milano, Italy) were added to the cervico-vaginal swabs, collected by a rigid cotton-tipped swab applicator (Nuova Aptaca, Cannelli, Italy), and vortexed. Subsequently, the samples were transferred to microcentrifuge tubes (2 ml) and they were stored at −20 °C until processing.

At processing moment, microcentrifuge tubes were centrifuged at rcf = 15 700 ***g*** for 15 min at 7 °C. The majority of supernatant was discarded but 200 µl of supernatant was retained to resuspend the pellet.

### DNA isolation and nested-PCR

From January 2012, DNA extraction was performed by automated QIAcube System (Qiagen, Germany) following the manufacturer's protocols. Extracted DNA samples were submitted to a nested PCR amplification, using an Ampliquality HPV-HS Bio Kit (AB Analitica, Padova, Italy) following the manufacturer's instructions.

The method provides for a first amplification of the viral genome L1 region, followed by a second amplification phase with biotinylated primers. PCR products were analysed using 3% agarose gel electrophoresis with ethidium bromide to display DNA under ultraviolet light. Subsequently, PCR products were typed by using a Reverse Line Blot Hybridisation Ampliquality HPV-Type Kit (AB Analitica, Italy). The Ampliquality HPV-Type Kit allows to detect 18 HR-HPVs types (types 16, 18, 26, 31, 33, 35, 39, 45, 51, 52, 53, 56, 58, 59, 66, 68, 73, 82) and 11 LR-HPVs types (types 6, 11, 40, 42, 43, 44, 54, 61, 70, 72, 81).

To assess the suitability of extracted DNA, thiosulfate sulfurtransferase (TST) gene region (202 bp) was amplified at the first amplification. A negative result TST amplification underlines the presence of PCR inhibitors or DNA degraded.

Moreover, the Ampliquality HPV-HS Bio Kit provides either one negative control or one positive control represented by plasmid DNA of HPV-54.

### DNA isolation and multiplex real-time PCR

From January 2014, viral nucleic acids were extracted from the resuspended pellet using the automated MagNa Pure 96 system™ (Roche Diagnostics GmbH, Mannheim, Germany) according to the manufacturer's instructions.

DNA extraction was carried out starting from 200 µl of sample. DNA was eluated in a final volume of 100 µl. Extracted DNA samples were submitted to multiplex real-time PCR (mRT-PCR) by an Anyplex™ II HPV 28 Detection System (Seegene, Seoul, Korea) performed on a CFX96 Real-Time PCR (Bio-Rad, Hercules, CA, USA). The ANYPLEX II HPV-28 Detection kit simultaneously detects 19 HR-HPV types (types 16, 18, 26, 31, 33, 35, 39, 45, 51, 52, 53, 56, 58, 59, 66, 68, 69, 73 and 82) and nine LR-HPV types (types 6, 11, 40, 42, 43, 44, 54, 61 and 70) from a single specimen using Tagging Oligonucleotide Cleavage and Extension (TOCE) technology. Moreover, the kit provides either one negative control or three positive controls for each of the two PCR reactions (panels A and B). Panel A includes 14 HR/HPV-types, while panel B includes five HR and nine LR-types. Data recording and interpretation were automated with Seegene viewer software according to the manufacturer's instructions.

### Quality controls measures

Currently, in our laboratory, either intra-laboratory quality controls or inter-laboratory quality controls (VEQ) have been defined and activated. In the case of intra-laboratory quality controls, previously analysed samples are used to in order to evaluate the results obtained for a long time. With reference to the inter-laboratory quality controls, we currently perform a commercially available VEQ program (DicoCARE VEQ).

### Statistical analysis

The association of the DNA extraction methods, PCR assays, sample types, countries of women with either HPV prevalence or mHPV infection was evaluated by the *χ*^2^ test, as appropriate.

Only the viral types 16, 18, 31, 33, 35, 39, 45, 51, 52, 53, 56, 58, 59, 66, 68, 73, 82, 6, 11, 40, 42, 43, 44, 54, 61, 70 were retained for the analysis because common to both the PCR assays used. In addition, HPV-26 was not retained because it had a too low frequency (three samples).

To assess the association among each pairing of HPV types, multivariate logistic regression was used. In particular, predictor variables (viral types, PCR assay, extraction method and nationality) were decided *a priori*. Missing ages were imputed by multiple imputation by fully conditional specification implemented in the Multivariate Imputation by Chained Equations (MICE) package implemented in environment R. Predictive mean matching imputation model was specified on the assumption of missing at random ages and the number of iterations was set to 20. In particular, 50 imputed datasets were generated. For each dataset, a logistic regression model was generated and the 50 models were pooled together by the function *pool* of the package mice. Globally, 26 logistic regression models were evaluated.

To simplify the final logistic regression models, nested models with and without extraction method and nationality variables were compared by likelihood ratio tests and the collected *P*-values were corrected by Benjamini and Hochberg's (BH) procedure with false discovery rate (FDR) <1% [23].

Finally, all *P*-values collected from the final simplified logistic regression models were corrected for multiple comparison by BH procedure with FDR < 1% .

Calculations of all statistical tests were performed using the open source environment R [24].

## Results

From January 2012 to February 2018, 2848 cervico-vaginal swabs from 2848 women were analysed. Median age of analysed women was 37 years (interquartile range (IQR): 29–45).

In total, 135/2848 (4.74%) women were not born in Italy. The prevalence of HPV infection in Italian and non-Italian women was 47.62% (1292) and 48.14% (65), respectively (*P-*value = 0.957) and the prevalence of mHPV infection was 24.36% (661) and 24.44% (33) (*P-*value = 1), respectively.

In total, 1357/2848 (47.65%) samples were positive, whereas 1491/2848 (52.48%) resulted negative for HPV DNA. In 694/1357 (51.14%) samples mHPV genotypes were detected. The median number of the HPV-types in mHPV infections was 2 (IQR: 2–3), ranging from a minimum of 2 (369 samples) to a maximum of 10 (one sample). In particular, we have found 369 (12.96%) and 164 (5.76%) women with two and three viral types, respectively.

From January 2012 to the end of June 2013, 414 samples were extracted by QIAcube System (Qiagen) followed by a nested PCR technique. From July 2013 to the end of December 2013, 349 specimens were extracted by QIAcube followed by mRT-PCR. From January 2014 to February 2018, 2085 samples were extracted by the MagNa Pure 96 system (Roche) and amplified by mRT-PCR. The HPV prevalences in the three groups were 44.69% (185), 50.43% (176) and 47.80% (996), respectively (*P-*value = 0.279). On the other hand, the mHPV infections were 16.42% (68), 25.50% (89) and 25.76% (537), respectively (*P-*value *<* 0.001) (Table 1).

**Table 1.** HPV prevalence stratified according to the different combinations of extraction and amplification methods

Extraction	Amplification	Total number	HPV *n* (%)	*P-*value	Multiple HPV *n* (%)	*P-*value
Qiagen	Nested-PCR	414	185 (44.69)	0.279	68 (16.42)	<0.001
Qiagen	mRT-PCR	349	176 (50.43)		89 (25.50)	
Roche	mRT-PCR	2085	996 (47.80)		537 (25.75)	

*P*-values were calculated on a 2 × 3 matrix by the *χ*^2^ test.

**Fig. 1.**
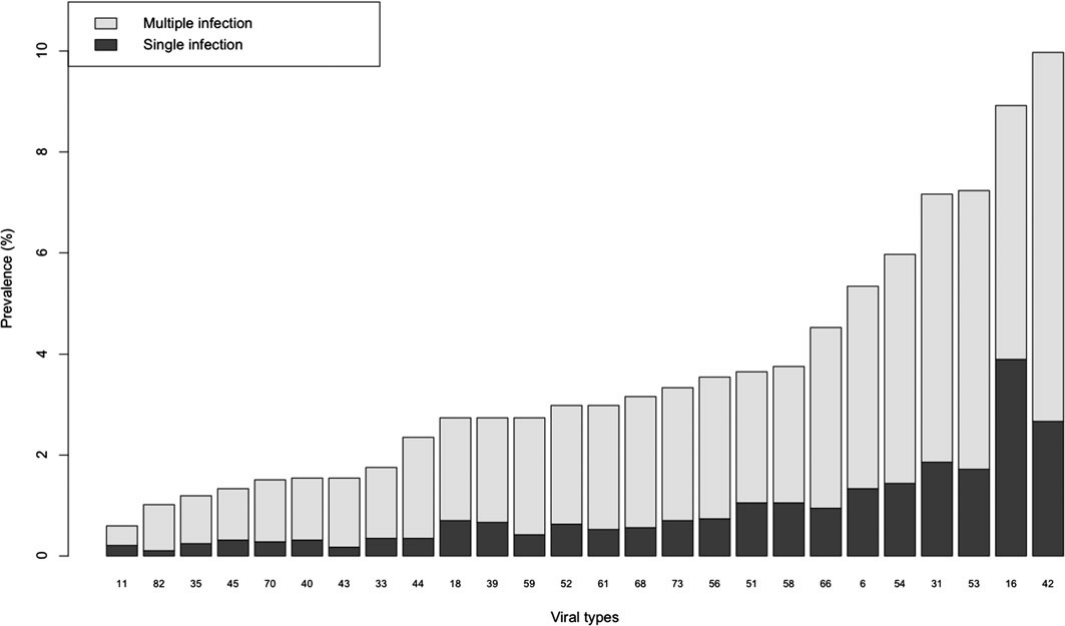
Type-specific infection prevalence in the 2848 analysed women.

The viral types more frequently detected were 42 (284/2848 samples, 9.97%) followed by 16 (254, 8.92%), 53 (206, 7.23%) and 31 (204, 7.16%), while those less frequently detected were 11 (17, 0.60%), 82 (29, 1.02%) and 35 (34, 1.19%). Each HPV-type was involved in mHPV infections in more than 50% of cases (Fig. 1). In particular, in the mHPV infections, the 42, 16, 53 and 31 prevalence was 7.30%, 5.02%, 5.51% and 5.30%, respectively, while the prevalence of 11, 82 and 35 was 0.39%, 0.91% and 0.95%, respectively.

**Fig. 2.**
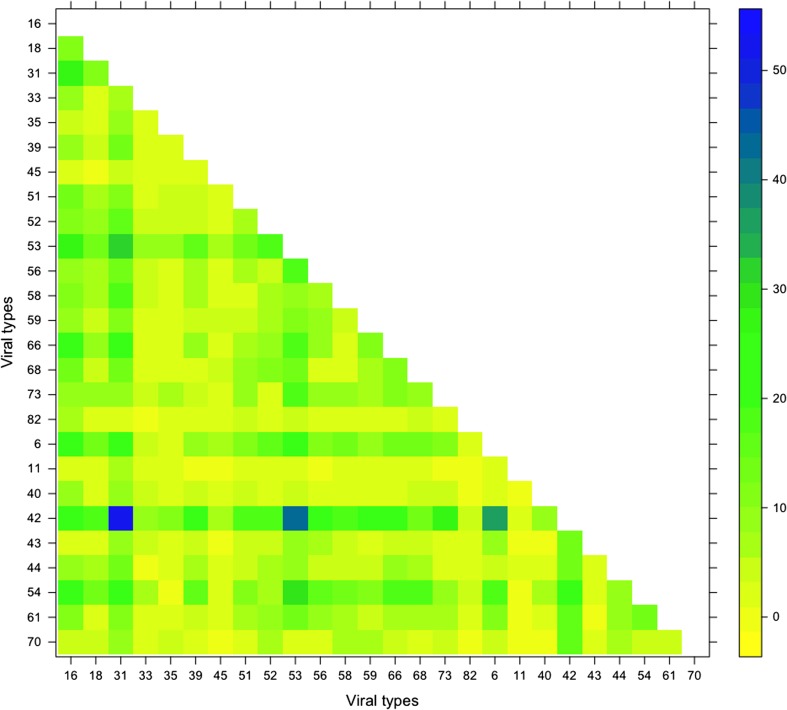
Frequency of pairwise of the viral types detected in the 2848 analysed women.

From the pairwise comparison of the viral types it is possible to note that the majority of the cases of two-type combinations involved 42, 54 and 31. In particular, the most frequently reported two-types combinations were 42/31 (52, 1.83%), 42/53 (42, 1.47%), 42/6 (36, 1.26%), 53/31 (32, 1.12%) and 54/53 (30, 1.05%) (Fig. 2).

The comparison of the 26 logistic regression models containing the variables age, nationality (Italian *vs.* non-Italian), PCR assay (mRT-PCR *vs.* nested PCR) and extraction method (Qiagen *vs.* MagnaPure 96) with the reduced models without the extraction method and nationality variables after BHs correction showed that all *P*-values were non-significant. For this reason the extraction method and nationality variables were not inserted in the 26 final models (data not shown).

The logistic regression analysis highlighted several statistically associated HPV combinations. Odds ratios are reported in Table 2 and the statistically significant associations after BHs correction have been highlighted in yellow colour. In particular, viral types 42 and 70 were involved in the major number of multiple positive associations. On the contrary, types 33, 43, 51, 58 and 82 were not involved in any statistically significant associations while 11, 16, 18, 40, 56 and 61 were involved in only one positive association. Negative associations were not detected by the regression analysis (Table 2).

**Table 2.** Association between HPV types by multivariate logistic regression models after *P*-values correction by BHs procedure

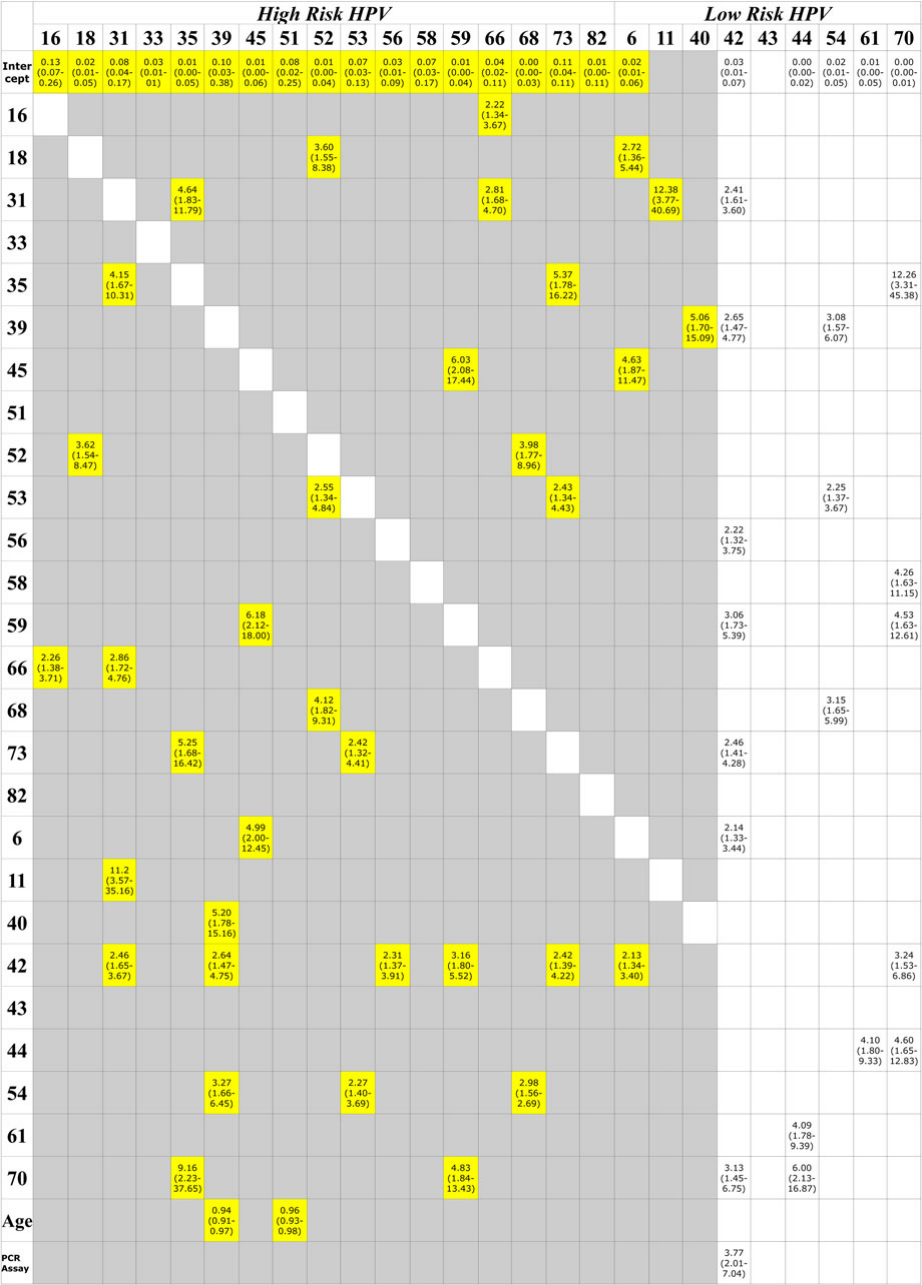

Strength of associations is expressed by the odds ratios and 95% confidence interval. Statistically significant associations are highlighted in yellow. The multivariate logistic regression models are reported on the 26 columns, while the covariants on the 29 lines.

Age: linear variable.

PCR assay: mRT-PCR *vs*. nested-PCR.

**Fig. 3.**
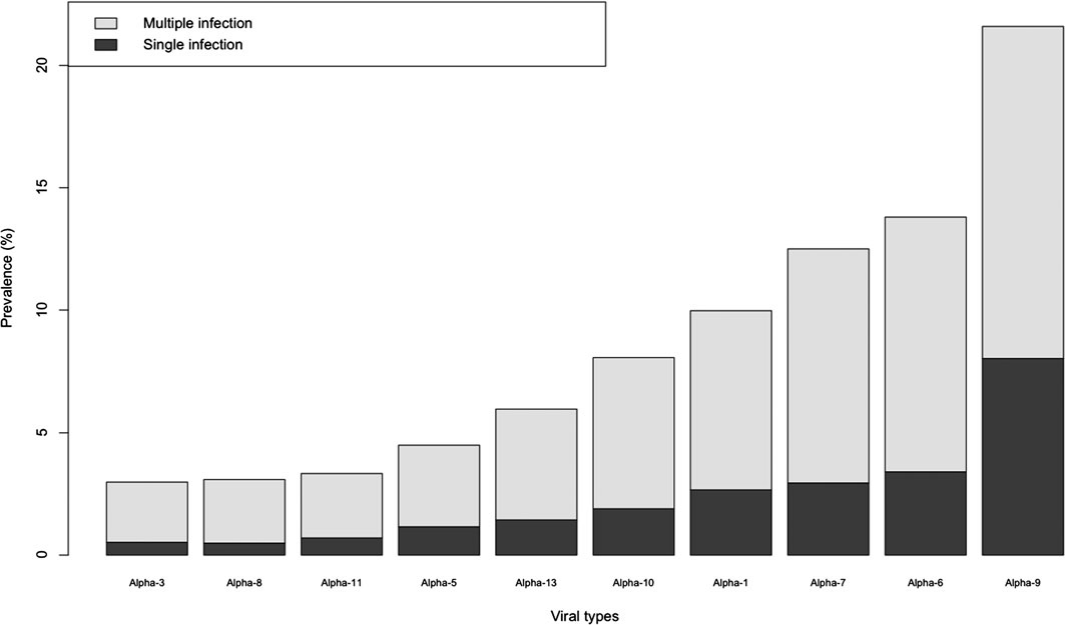
Phylogenetic-specific infection prevalence in the 2848 analysed women.

The most common phylogenetic group was *α*−9 (615/2848, 21.59%), followed by *α*−6 (393/2848, 13.80%) and *α*−7 (356/2848, 12.50%) (Fig. 3). All phylogenetic groups were involved in positive associations (Table 3).

**Table 3.** Association between HPV phylogenetic groups by multivariate logistic regression models after *P*-values correction by BHs procedure

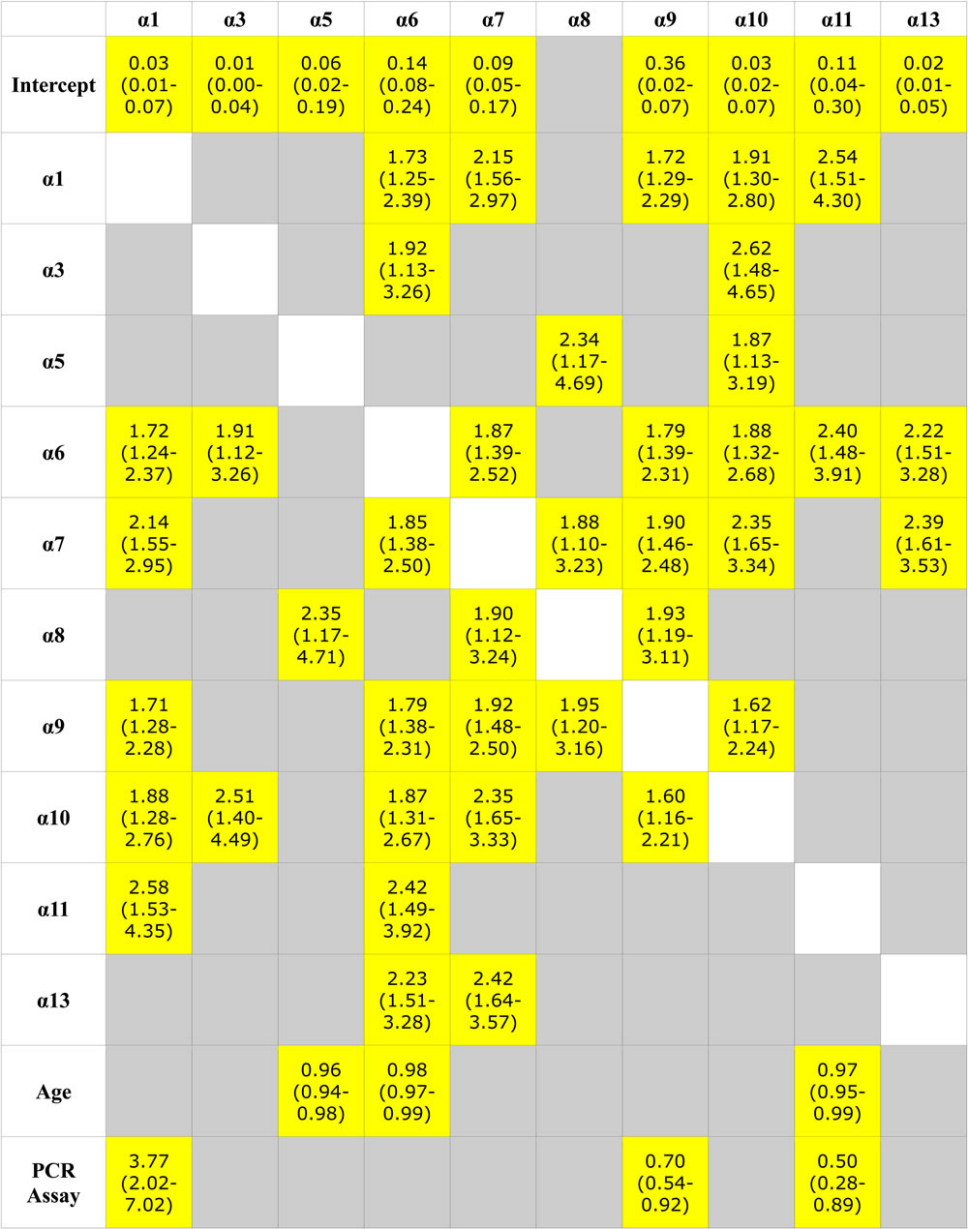

Strength of associations is expressed by the odds ratios and 95% confidence interval. The multivariate logistic regression models are reported on the 10 columns, while the covariants on the 13 lines. Statistically significant associations are highlighted in yellow.

Age: linear variable.

PCR Assay: mRT-PCR *vs*. nested-PCR.

## Discussion

Although HPV is considered the major causative agent for CC, little is known about the additional factors as the association among the pattern of HPV-types co-infections and the CC. To date, only few studies have reported a relationship between mHPV infections and CC, but the results obtained are not conclusive. Some studies have suggested a possible role of mHPVs in the development of CC [22, 25–28], while others works have reported that the risk of CC in women infected with mHPVs was no greater than that with single-type infections [29, 30].

Our study investigates the prevalence of HPV-types in multiple infections. In many works, the differences observed when the prevalences of HPV infections had been studied, were due to several factors as geographic parameters, age, sexual behaviour, variables affecting immune response and HPV detection methods [29, 31]. Similarly, the prevalence of mHPV infections also ranged significantly from 1% to 20%, due to the HPV detection methods used [29, 31, 32]. On basis of different extraction and amplification assays performed, we did not found significant differences in HPV prevalences (44.69% *vs.* 50.43% *vs.* 47.80%).

In agreement with Dickson *et al*. [33], our study suggests that mHPV infections frequently occurred (51.14%). In particular, the most common HR-HPV-type that we have detected was HPV-16. According to Oliveira *et al*. [34] and Chatuverdi *et al*. [22], HPV-16 is involved less frequently in single infections rather than in mHPV infections. Other HR-HPV-types identified more frequently in single infections are 53 (7.23%) and 31 (7.16%). Our results are in accordance with other epidemiological studies [35–38]. Moreover, only our study shows that 53 and HPV-31 (HR-HPV) together with 42 (LR-HPV) are the most representative HPV-types involved in co-infections. In addition, we have detected the 42 as the most prevalent HPV-type in either single or mHPV infections.

When the prevalence of combinations was studied, the most representative two-types combinations reported were 42/31, 42/53, 42/6, 53/31 and 54/53. On the contrary, Aleksioska-Papestiev *et al*. [39] have reported HPV16/18 and HPV 16/31 as the most common combinations of mixed HPV infection.

Moreover, Mori *et al*. [40] studying on the replication interference between HPV-16 and 18 have shown that these genotypes, when co-infected alone in absence of other HPV-types, might decrease the risk of CC, explaining their antagonistic action. However, in our study, we did not found any associations among HPV-16 and HPV-18, in agreement with Dickson *et al*. [33].

In addition, our study shows that all phylogenetic groups are involved in positively associated combinations, but only the HPV-types belonging to *α*−9, *α*−6 and *α*−7 species are the most prevalent in two-type combinations. On the contrary, these results are not supported by the studies of Trottier *et al*. [32] and Dickson *et al*. [33], which have described the *α*−9 species as the only involved in mHPV infections.

There are several limitations of this study. First, the reported data are not representative of the overall Apulia population so the results are not generalisable. A major limitation is the absence of the cytological data on the enrolled individuals. So, not having the data of histology of cervical lesions, it was not possible to correlate the presence of HPV with the cytological and/or histological findings and it was not possible to argue regarding the role of the mHPVs in the progression of the lesions. Moreover, the lack of behavioural information on the enrolled individuals did not permit to better characterise the analysed population for the roles of such exposure variables in the diffusion of HPV infections, therefore, we have not be able to analyse the association between these aetiological factors and HPV prevalence. Moreover, because of the presence of the same acquisition way, the effect estimate of measures of association between sexually transmitted infections may be confounded by common risk factors.

On the other hand, this study accounted for the analytical methods used to extract and genotype HPV. In fact, the two DNA extraction methods were not found associated with any HPV-types while the pairwise mHPVs detected were controlled for the two genotyping assays. To our knowledge, the evaluation of the analytical methods as confounding factor for mHPV associations has been object of very few studies [1].

In conclusion, the impact of HPV co-infections on the clinical outcome is still not clear. However, it is likely that only specific HPV combinations may be associated with a significant clinical impact while other combinations may simply be correlated because of the common infection route or to the diagnostic method used. In particular, some specific combinations could synergistically or antagonistically interact and subsequently affect the risk of development of CC. Therefore, further clinical studies are needed either to determine the mechanism of these mHPV infections or the clinical outcome related to particular combinations of co-infected HPV-types.
